# Molecular diversity and relationships of fig associated nematodes from South Africa

**DOI:** 10.1371/journal.pone.0255451

**Published:** 2021-08-10

**Authors:** Meike S. Kruger, Natsumi Kanzaki, Robin M. Giblin-Davis, Jaco M. Greeff

**Affiliations:** 1 Department of Biochemistry, Genetics and Microbiology, University of Pretoria, Pretoria, South Africa; 2 Kansai Research Centre, Forestry and Forest Products Research Institute, Kyoto, Japan; 3 Department of Entomology and Nematology, Fort Lauderdale Research and Education Centre, University of Florida/IFAS, Davie, FL, United States of America; Laboratoire de Biologie du Développement de Villefranche-sur-Mer, FRANCE

## Abstract

Nematodes of figs and fig wasps have received limited attention in Africa since their discovery in 1973. Sixteen of the 25 species of native South African figs were sampled for nematode associates using molecular barcoding with three loci (SSU, LSU D2-D3 and mtCOI) and fourteen (93%) were positive for at least one nematode species. Thirty-three putative species of nematodes were identified and classified according to the loci that were amplified and successfully sequenced. Fourteen putative nematode species were classified as Aphelenchoididae, of which nine were identified as *Ficophagus* from four species of *Ficus* from the section *Galoglychia* (i.e., five ex *F*. *burkei* including one shared with *F*. *natalensis*, one ex *F*. *glumosa*, one ex *F*. *lutea*, and one ex *F*. *stuhlmannii*) and one species ex *F*. *sur* from the section *Sycomorus*. In addition, there were four nematode species classified as *Schistonchus* s.s. from section *Galoglychia* figs (i.e., one ex *F*. *burkei*, two ex *F*. *trichopoda*, and one ex *F*. *glumosa*). There was also one species of *Bursaphelenchus* nematode recovered from *F*. *sur* from the section *Sycomorus*. Sixteen putative nematode species were classified as Diplogastridae, of which eight occurred in two clades of what is currently called *Parasitodiplogaster* with one (*P*. *salicifoliae*) being recovered from two *Ficus* species in the section *Urostigma* (*F*. *salicifolia* and *F*. *ingens*) and seven diplogastrids being associated with six species of *Ficus* from the section *Galoglychia* (i.e., two ex *F*. *burkei* including *P*. *sycophilon*, one ex *F*. *stuhlmannii*, one ex *F*. *burtt-davyi*, one ex *F*. *trichopoda*, one ex *F*. *abutilifolia* and one ex *F*. *sansibarica*). Three *Acrostichus* spp., a *Teratodiplogaster* and a *Pristionchus* species were recovered from *F*. *sur* and two *Teratodiplogaster* spp. and *Pristionchus sycomori* were recovered from *F*. *sycomorus* with both *Ficus* species belonging to the subgenus and section *Sycomorus*. The identities of the previously described *T*. *martini* and *Parasitodiplogaster doliostoma* (= *Pristionchus* sp. 35) are discussed. Lastly, there was a panagrolaimid identified from *F*. *petersii*.

## Introduction

Martin et al. [1] first reported about the amazing morphological diversity of nematodes inside figs from six native species of *Ficus* from Zimbabwe in 1973 and invited participation from the global nematological community to help elucidate their identities and biology. Because non-native (introduced) figs were reported to be without fig wasp pollinators and did not have any nematode associates, a tight ecological and evolutionary relationship between nematodes, fig wasps, and figs was hypothesized [1]. Even though a few nematodes have been described from Africa, work elsewhere has shown that nematodes from two families, the Aphelencoididae and Diplogastridae, are frequently associated with figs [2]. In general, members of the Diplogastridae can have useful typological characters for species/generic separations whereas members of the Aphlenchoididae often do not [3, 4], but cryptic species are possible within both groups [5–7]. Furthermore, diverse phenotypes can be produced by a single genotype in some members of both families [8–11]. For instance, Susoy et al. [8] reported how a diverse array of up to 5 phenotypes can be manifested by a single new species of *Pristionchus* (i.e., *P*. *sycomori* Susoy, Kanzaki, Kruger, Ragsdale & Sommer in Susoy et al. and *Pristionchus* sp. 35) in sycones of *F*. *sycomorus* L. and *F*. *sur* Forsskål in South Africa, respectively.

While both nematode families have been described from a *Ficus* in the African section/subsection *Galoglychia* [12, 13], it is not clear if this pattern is common in Africa. *Galoglychia* and three of the other five sections of *Ficus* that occur in Africa, *Sycidium*, *Sycomorus* and *Urostigma* reach right down to South Africa [14]. Subsections of these three sections also occur in Asia, Southeast Asia and Australia where both nematode lineages have been found [14].

Poinar [12] described the first new species/genus of fig/fig wasp nematode from Africa in 1979, i.e., *Parasitodiplogaster sycophilon* Poinar (Diplogastridae) from the fig wasp, *Elizabethiella stuckenbergi* Grandi (Agaonidae) in figs (sycones) of *Ficus burkei* (Miq.) from Zimbabwe. Vovlas et al. [13] described *Schistonchus africanus* Vovlas, Troccori, van Noort & van den Berg (Aphelenchoididae) from figs of *F*. *burkei* and the fig wasp *E*. *stuckenbergi* from South Africa. Kanzaki et al. [15] described *Teratodiplogaster martini* Kanzaki, Giblin-Davis, Davies & Center (Diplogastridae) from *Ficus* sp. and *Parasitodiplogaster doliostoma* Kanzaki, Giblin-Davis, Davies & Center from *F*. *sur* from fixed materials provided from Martin et al.’s [1] original collections, and Jauharlina et al. [16] outlined the association dynamics between *Parasitodiplogaster* sp. and its fig wasp host *E*. *baijnathi* Wiebes and sycones of *F*. *burtt-davyi* Hutchinson from South Africa. More recently, Wöhr et al. [17, 18] re-described *Parasitodiplogaster sycophilon* from *F*. *burkei* and described *Parasitodiplogaster salicifoliae* Wöhr, Greeff, Kanzaki & Giblin-Davis from figs of *F*. *salicifolia* Vahl */ F*. *ingens* (Miq.) from South Africa, respectively. Except for the most recent work [8, 17, 18], all African fig/fig wasp nematode taxonomy was done solely with traditional morphology, which can be misleading with certain nematode groups and requires molecular sequence data for placement of these taxa into a modern phylogenetic framework.

Since adult or juvenile nematodes are phoretic/necromenic/or parasitic on the species-specific fig wasps and fossil evidence suggests that *Parasitodiplogaster*’s relationship is at least 19 million years old and they may well have co-diversified with the pollination mutualism [19] we can expect these two families to frequent figs from southern Africa. The paucity of information from African *Ficus* sections*/*subsections that occur on other continents and the phenotypic conservatism and occasional extreme trophic plasticity suggest a broad survey in Africa would be insightful. In the present study, we surveyed as many native figs as possible over a four-year period in South Africa to examine the identity and patterns of nematode associates of figs.

## Materials and methods

### Ethics statement

Specific permissions were not required for the nematodes collected for the present study. The trees sampled for nematode collection were either inside the University of Pretoria campus or places open to the public. Endangered or protected species were not involved with the present study.

### Nematode collection

**Fig 1:**
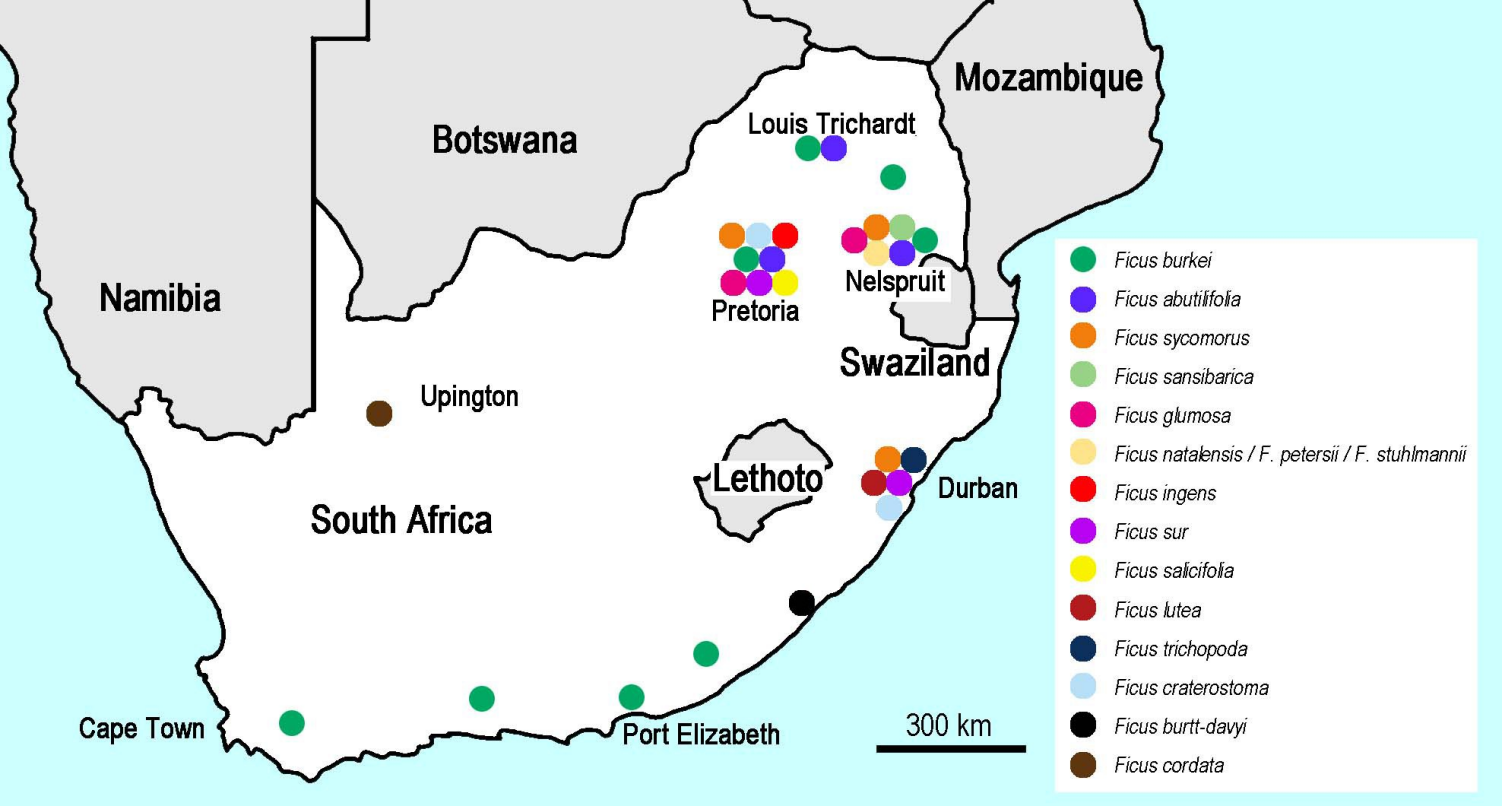
Map of southern Africa, country names in large font, city names in small font. Each color dot represents a fig species sampled in the area, mostly associated with cities. The color legend defines the fig species that each color represents.

Fig syconia in various phases (phases B-E) were collected from 25 *Ficus* species from many locations across South Africa (Fig 1, Table 1, S1 Table in S2 File). In general, fig syconia manifest phenologically in five phases based upon the pollination mutualism and corresponding development of the fig and its fig wasp pollinators as described by Janzen [20]. Because nematodes are vectored into the developing fig only after the syconia becomes receptive at phase B (female), phase A (pre-receptive or pre-female) figs were not used. In most cases, the ideal phases for nematode collection were early to late interfloral (phase C) or male (phase D) figs which were always used, if available. However, because fruit availability can be unpredictable, we collected phase B and E if it was the only fruit available. An average of 50 syconia were collected per crop and never less than 30. Collected figs were cut into *ca* 1–2 mm thick pieces and soaked in water for 10–15 min. Nematodes recovered from the sliced figs were hand-picked, identified to their taxonomic group at genus or family level at low magnification under a dissecting microscope according to body and stomatal/stylet shapes, and 3–5 individuals for each morphotypes were placed into either 100% ethanol or nematode digestion buffer (NDB) [21, 22], but less than that if there were insufficient specimens recovered. The rest of the individuals were killed by heat and fixed in formalin and processed into glycerin [23] as morphological vouchers. In addition, some individuals isolated from *F*. *sur* and *F*. *sycomorus* were transferred to DESS [24] to eventually compare their typological characters and molecular phylogenetic status for single nematodes.

**Table 1:** Host, locality and recovered nematode species from dissected fig syconia.

Fig section: subsection	*Ficus* species	Collection localities	Nematode species ^a^	Previously reported pollinator wasp species ^b^
*Galoglychia*: *Chlamydodorae*	*F*. *burkei*	Abel Erasmus pass, Phiphidi, Nzhelele Valley, Louis Trichardt, Hartbeespoort Dam	*Parasitodiplogaster sycophilon*, *Parasitodiplogaster* n. sp. 6, *Ficophagus* n. spp. 1, 2, 5, 6, 7, *Schistonchus* n. sp. 4	*Elisabethiella stuckenbergi*
	*F*. *natalensis*	Ballito	*Ficophagus* n. sp. 7	*Elisabethiella socotrensis*
	*F*. *burtt-davyi*	Eastern Cape	*Parasitodiplogaster* n. sp. 2	*Elisabethiella baijnathi*
	*F*. *petersii*	Nelspruit	Unidentified panagrolaimid^c^	*Alfonsiella binghami*
	*F*. *craterostoma*	Pretoria	Morphological vouchers only, not genotyped ^d^	*Alfonsiella pipithensis*
*Galoglychia*: *Platyphyllae*	*F*. *stuhlmannii*	Nelspruit	*Parasitodiplogaster* n. sp. 5, *Ficophagus* n. sp. 8	*Alfonsiella binghami*
	*F*. *abutilifolia*	Pretoria	*Parasitodiplogaster* n. sp. 3	*Elisabethiella comptoni*
	*F*. *trichopoda*	Ballito	*Parasitodiplogaster* n. sp. 1, *Schistonchus* n. spp. 1, 2	*Elisabethiella bergi breviceps*
	*F*. *glumosa*	Nelspruit, Pretoria	*Schistonchus* n. sp. 3	*Elisabethiella glumosae*
*Galoglychia*: *Galoglychia*	*F*. *lutea*	Ballito	*Ficophagus* n. sp. 9	*Allotrizoon heterandromorphum*
*Galoglychia*: *Caulocarpae*	*F*. *sansibarica*	Nelspruit	*Parasitodiplogaster* n. sp. 4	*Courtella armata*
*Urostigma*: *Urostigma*	*F*. *ingens*	Pretoria	*Parasitodiplogaster salicifoliae*	*Platyscapa soraria*
	*F*. *salicifolia*	Pretoria, Nelspruit	*Parasitodiplogaster salicifoliae*	*Platyscapa awekei*
	*F*. *cordata*	Augrabies Falls	Morphological vouchers only, not genotyped^d^	*Platyscapa desertorum*
*Sycomorus*: *Sycomorus*	*F*. *sur*	Ballito, Pretoria	*Teratodiplogaster* cf. *martini*, *Acrostichus* n. spp. 1, 2, 3 (and 4), *Pristionchus* sp. 35 ex Susoy et al. [8] (= cf. “*Parasitodiplogaster*” *doliostoma* ex Kanzaki et al. [15]), *Ficophagus* n. sp. 4	*Ceratosolen capensis*
	*F*. *sycomorus*	Pretoria, Nelspruit	*Teratodiplogaster* n. spp. 1, 2, *Pristionchus sycomori*	*Ceratosolen arabicus*

^a^ The numbers suggest the genotype code considered as different undescribed species based on the differences of molecular sequences.

^b^ Information from van Noort & Rasplus [14].

^c^ Not analyzed phylogenetically because only relatively short (ca 400 bps) fragment of D2-D3 LSU was obtained, and no close relative was found in GenBank, i.e., the closest sequences were *Macrolaimus* spp. with 80% of identity (see text).

^d^ Although multiple species of nematodes were found, molecular materials were not available, and only collection records are presented here.

### Molecular profiles and phylogeny

The nematodes transferred to 100% ethanol or NDB were used for molecular samples to survey the diversity of nematode genotypes associated with different fig species from different locations, i.e., DNA was extracted from ethanol-fixed material using a QIAamp ® DNA Micro Kit (Qiagen, Germany) and NDB-treated samples were digested under 55°C for 20 min. Whereas the DESS-fixed materials were re-hydrated in a drop of sterile water, photo-documented (see below for methods), transferred to NDB and digested for use as template DNA.

We attempted to amplify and sequence three genetic loci, i.e., nearly full-length small subunit (SSU) and D2-D3 expansion segments of the large subunit (LSU) of ribosomal RNA genes and a partial region of the mitochondrial cytochrome oxidase subunit I (mtCOI) for all DNA template samples. Methodologies of Ye et al. [25] and Kanzaki and Futai [26] were employed for sequencing ribosomal RNAs and mtCOI. Briefly, amplification of SSU, LSU and mtCOI were attempted with primer sets, SSUF07 (5’—AAA GAT TAA GCC ATG CAT G—3’) and SSUnR (5’—TTA CGACTT TTG CCC GGT TC—3’), D2a (5’—ACA AGT ACC GTG AGG GAA AGT TG—3’) and D3b (5’—TCG GAA GGA ACC AGC TAC TA—3’), and COIF1 (5’—CCT ACT ATG ATT GGT GGT TTT GGT AAT—3’) and COIR2 (5’—GTA GCA GTA AAA TAA GCA CG—3’), respectively. Upon successful amplification, sequences were determined with Sanger sequencing using the purified amplicons. Sequences with ambiguous or poor quality electropherogram/chromatograms were omitted. The sequences were compared with those deposited in the GenBank database using BLASTN program (https://blast.ncbi.nlm.nih.gov/Blast.cgi?PROGRAM=blastn&PAGE_TYPE=BlastSearch&LINK_LOC=blasthome) for initial identification, and the generic status of all compared sequences were confirmed by their phylogenetic status. High quality sequences are summarized in Table 1 and S1 Table in S2 File.

**Fig 2:**
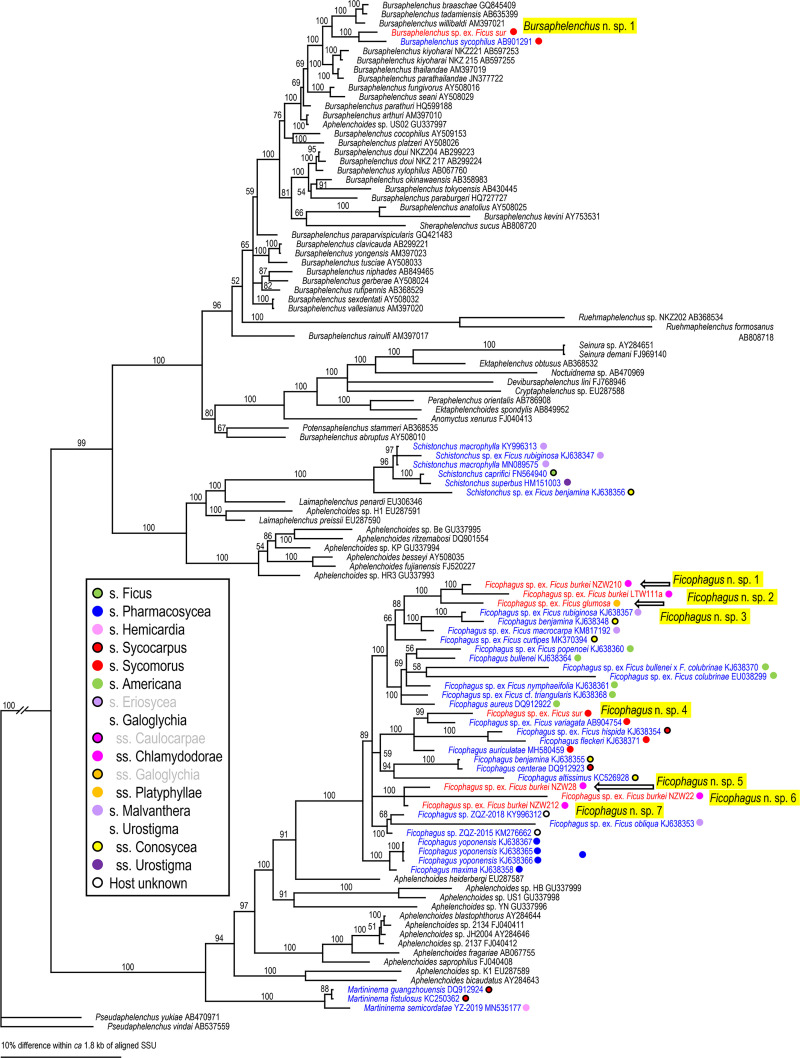
Phylogenetic status of fig-associated species within the family Aphelenchoididae inferred from SSU under GTR + G model. The parameters are: AIC = 56880.841, lnL = -28210.097, freqA = 0.26, freqC = 0.20, freqG = 0.27, freqT = 0.27, R(a) = 0.85, R(b) = 2.47, R(c) = 1.02, R(d) = 0.88, R(e) = 3.48, R(f) = 1.00, Pinva = n/a, Shape = 0.39. Posterior probability values exceeding 50% are given on appropriate clades. Fig-associated nematode species obtained in the present study and previous studies are written in red and blue, respectively. Section or subsection of host fig species are coded with color dots.

**Fig 3:**
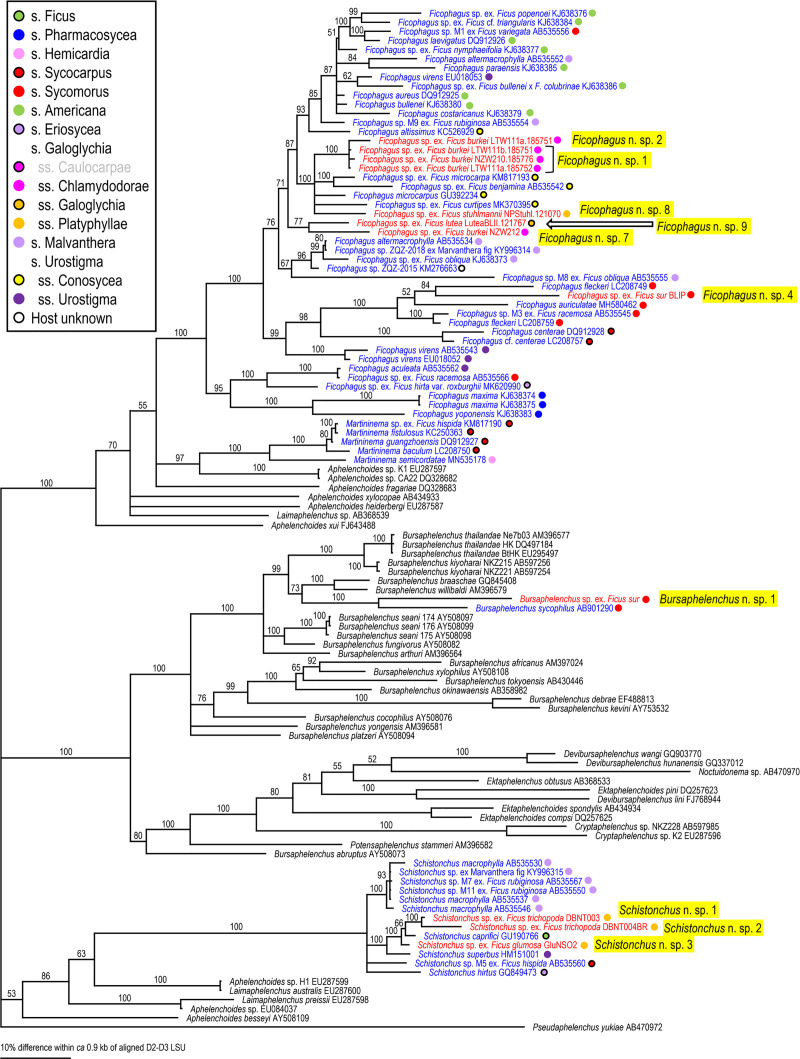
Phylogenetic status of fig-associated species within the family Aphelenchoididae inferred from D2-D3 LSU under GTR + G + I model. The parameters are: AIC = 55193.907, lnL = -27357.223, freqA = 0.24, freqC = 0.19, freqG = 0.30, freqT = 0.27, R(a) = 0.45, R(b) = 2.61, R(c) = 0.92, R(d) = 0.64, R(e) = 3.31, R(f) = 1.00, Pinva = 0.11, Shape = 0.86. Posterior probability values exceeding 50% are given on appropriate clades. Fig-associated nematode species obtained in the present study and previous studies are written in red and blue, respectively. Section or subsection of host fig species are coded with color dots.

**Fig 4:**
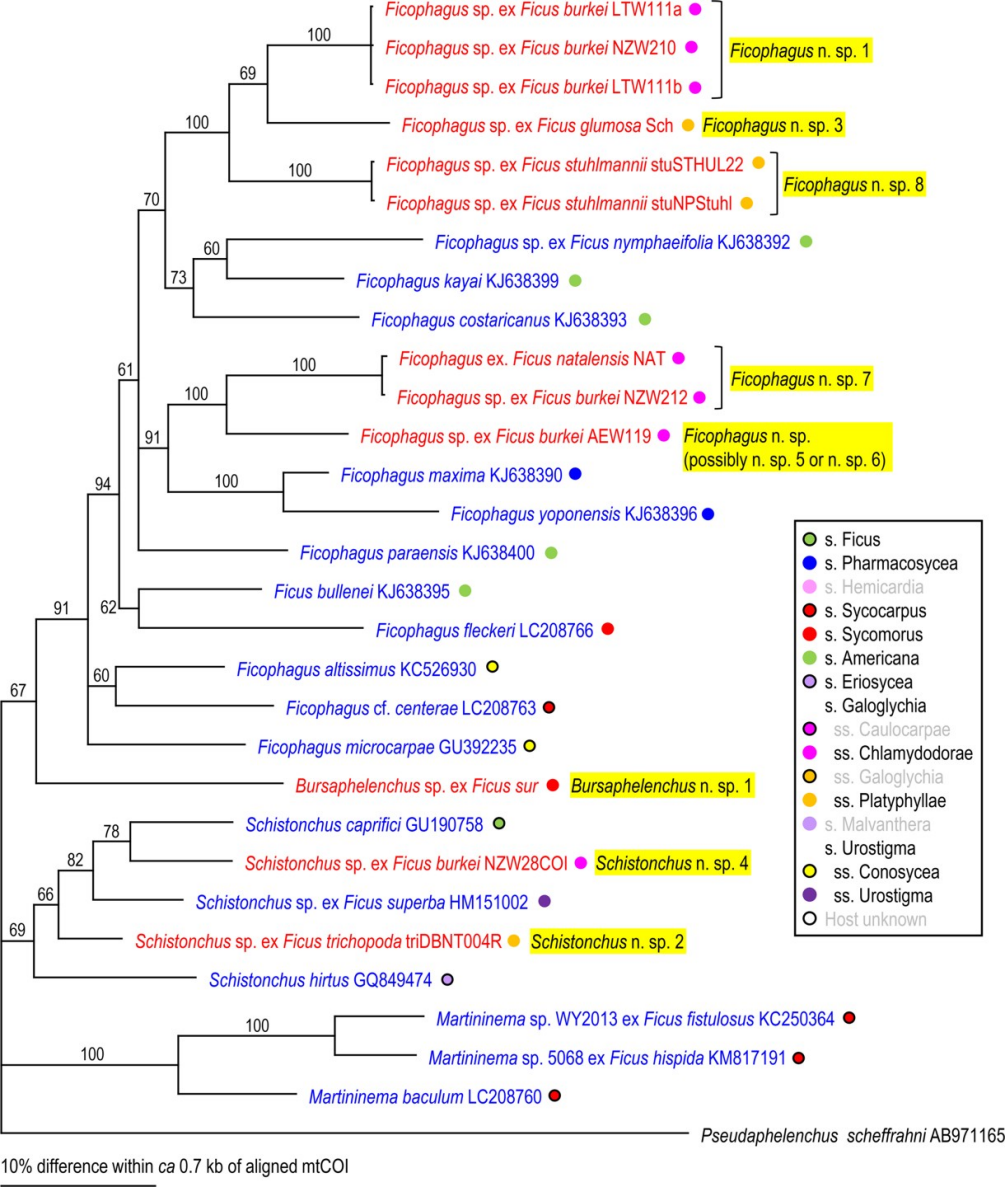
Phylogenetic relationship of fig-associated Aphelenchoididae species inferred from mtCOI under GTR+G+I model. The parameters are: AIC = 13195.267, lnL = -6530.39, freqA = 0.22, freqC = 0.12, freqG = 0.20, freqT = 0.46, R(a) = 0.07, R(b) = 2.45, R(c) = 1.17, R(d) = 0.49, R(e) = 2.12, R(f) = 1.00, Pinva = 0.44, Shape = 1.04. Posterior probability values exceeding 50% are given on appropriate clades. Fig-associated nematode species obtained in the present study and previous studies are written in red and blue, respectively. Section or subsection of host fig species are coded with color dots.

**Fig 5:**
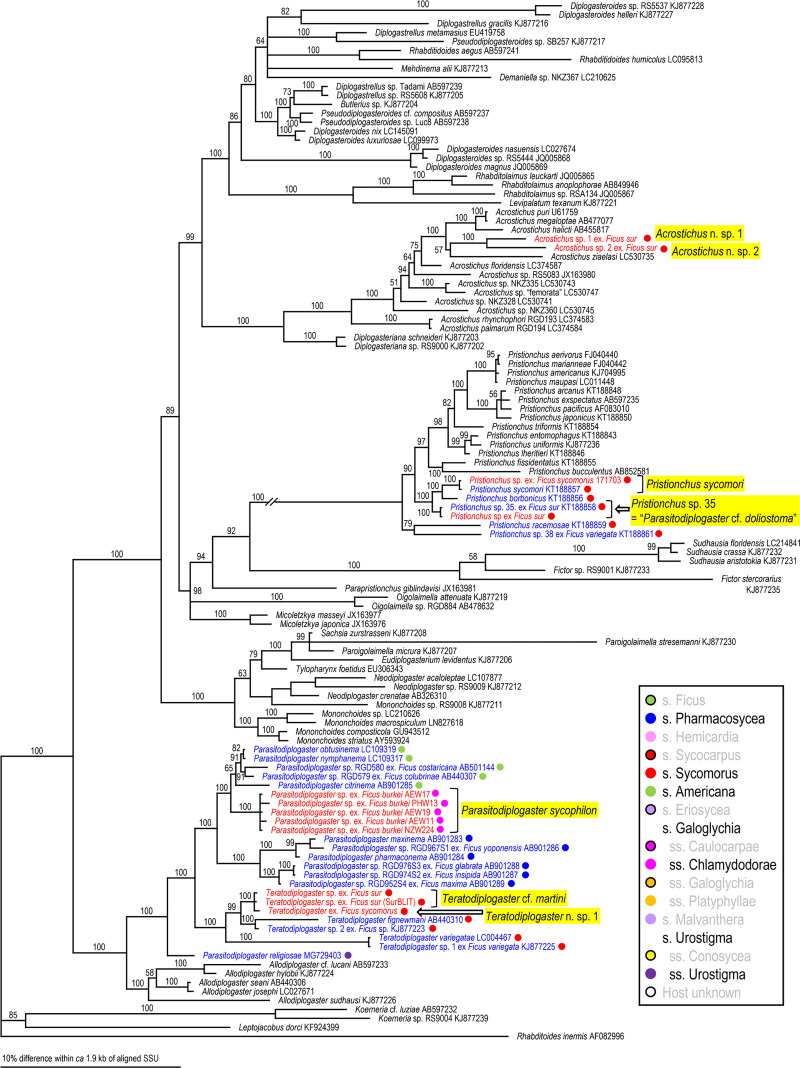
Phylogenetic status of fig-associated species within the family Diplogastridae inferred from SSU under GTR + G + I model. The parameters are: AIC = 50531.581, lnL = -25026.463, freqA = 0.25, freqC = 0.21, freqG = 0.27, freqT = 0.27, R(a) = 0.94, R(b) = 2.60, R(c) = 2.10, R(d) = 0.92, R(e) = 3.80, R(f) = 1.00, Pinva = 0.35, Shape = 0.53. Posterior probability values exceeding 50% are given on appropriate clades. Fig-associated nematode species obtained in the present study and previous studies are written in red and blue, respectively. Section or subsection of host fig species are coded with color dots.

**Fig 6:**
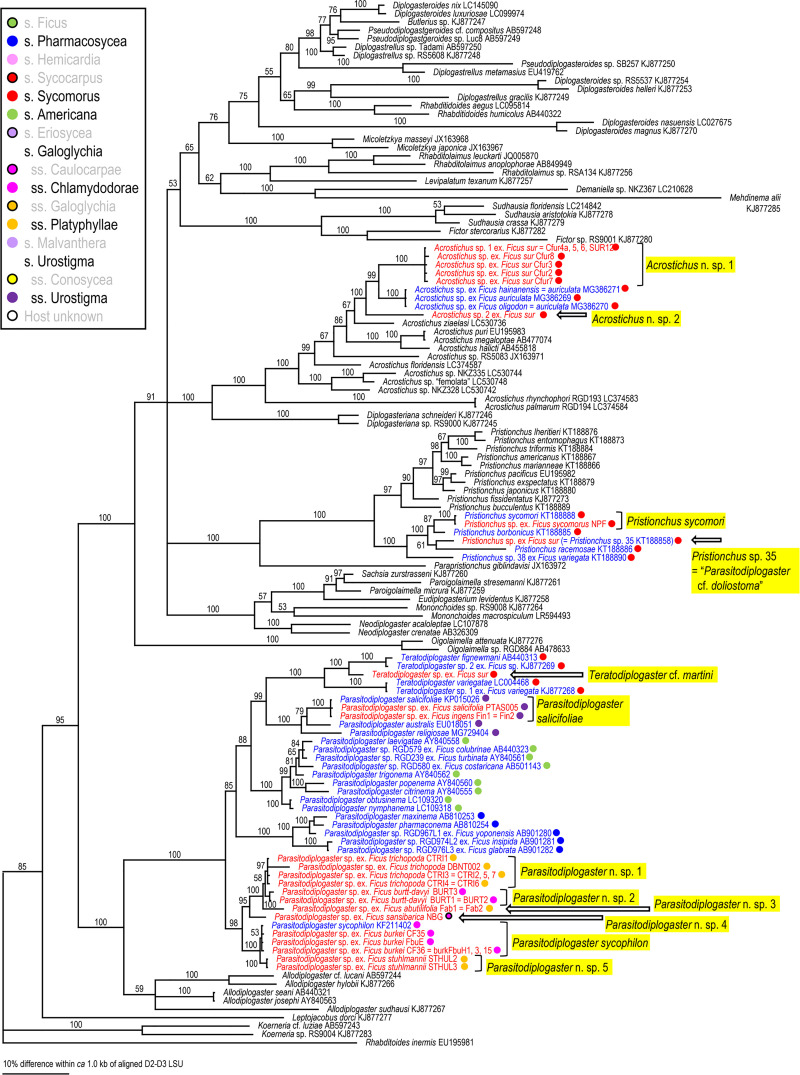
Phylogenetic status of fig-associated species within the family Diplogastridae inferred from D2-D3 LSU under GTR + G + I model. The parameters are: AIC = 55649.066, lnL = -27559.395, freqA = 0.20, freqC = 0.22, freqG = 0.33, freqT = 0.25, R(a) = 0.53, R(b) = 1.92, R(c) = 0.89, R(d) = 0.50, R(e) = 3.51, R(f) = 1.00, Pinva = 0.21, Shape = 0.94. Posterior probability values exceeding 50% are given on appropriate clades. Fig-associated nematode species obtained in the present study and previous studies are written in red and blue, respectively. Section or subsection of host fig species are coded with color dots.

**Fig 7:**
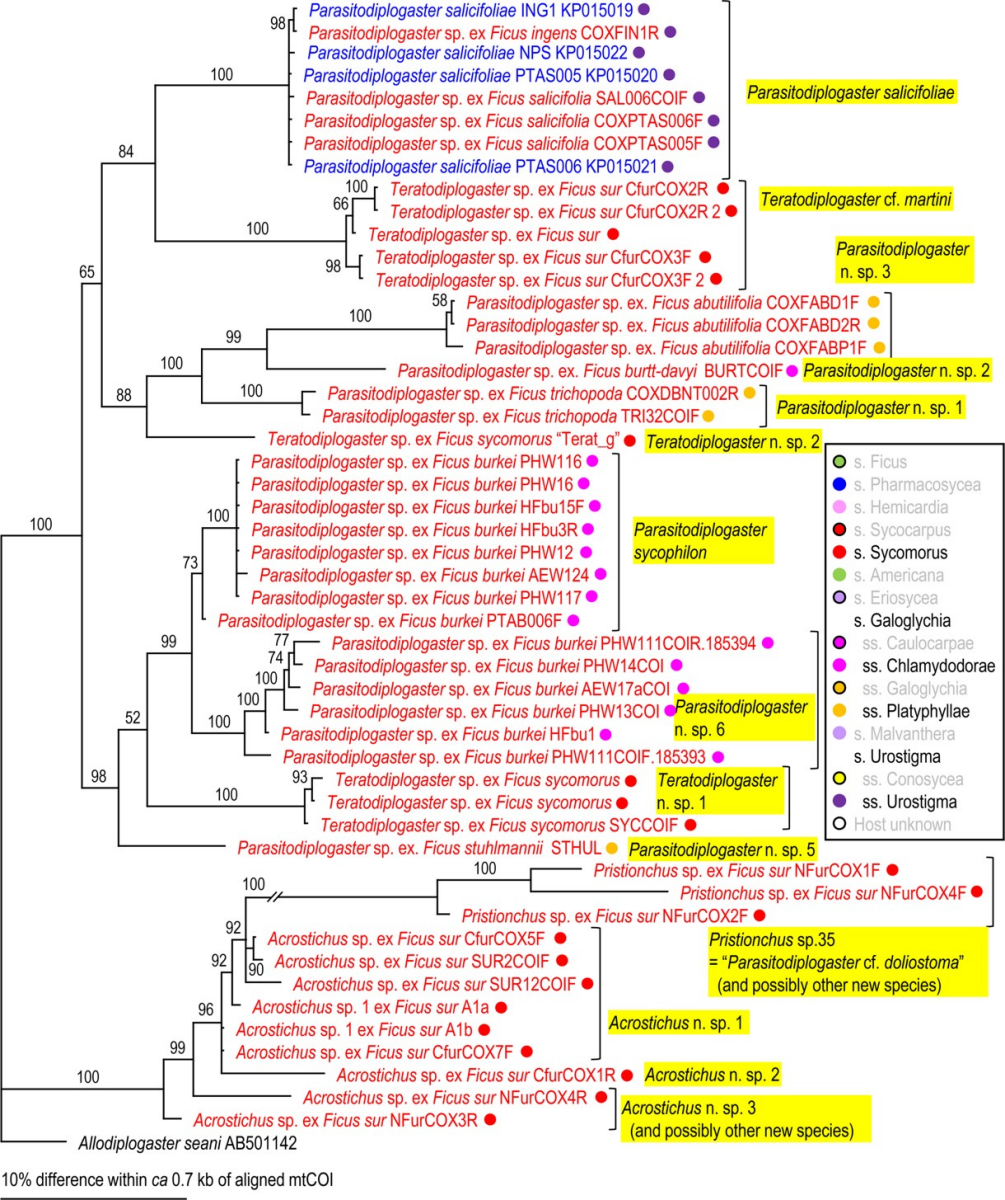
Phylogenetic relationship of fig-associated Diplogastridae species inferred from mtCOI under GTR + G model. The parameters are: AIC = 9938.549, lnL = -4858.882, freqA = 0.26, freqC = 0.12, freqG = 0.19, freqT = 0.43, R(a) = 0.44, R(b) = 3.03, R(c) = 1.72, R(d) = 0.68, R(e) = 3.24, R(f) = 1.00, Pinva = n/a, Shape = 0.32. Posterior probability values exceeding 50% are given on appropriate clades. Fig-associated nematode species obtained in the present study and previous studies are written in red and blue, respectively. Section or subsection of host fig species are coded with color dots.

Because two groups (= families), Aphelenchoididae and Diplogastridae were recognized, these families were phylogenetically analyzed separately for each locus using Bayesian Inference (BI) to determine their phylogenetic status within family (SSU and D2-D3) or other fig-associates (mtCOI). Compared sequences and their GenBank accession numbers are shown in Figs 2–7. The compared sequences were selected according to previous family-wide analyses, i.e., Kanzaki et al. [27] and Davies et al. [3] for Aphelenchoididae and Wöhr et al. [17], Zeng et al. [28] and Gonzalez et al. [29] for Diplogastridae.

Compared sequences were aligned using the MAFFT program [30, 31] (https://mafft.cbrc.jp/alignment/server/index.html) with the default settings. The base-substitution models for each gene were determined using the MEGA 7 software [32] and the Akaike information criterion. Combined Bayesian analysis was performed using the MrBayes 3.2 software [33, 34]; four chains were run for 4 × 10^6^ generations, and Markov chains were sampled at intervals of 100 generations [35]. Two independent runs were performed, and after confirming convergence and discarding the first 2 × 10^6^ generations as ‘burn in’, the remaining topologies were used to generate a 50% majority-rule consensus tree.

### Morphological observation

Several DESS-fixed materials were rehydrated and observed for their typological characters, particularly stomatal/stylet and pharyngeal morphologies and male and female tail characters which are usually used for diagnostic characters, prior to digestion. The materials were temporarily mounted on an agar pad, observed under a light microscope (Eclipse 80i, Nikon) with differential interference contrast (DIC) optics and photomicrographed using a microscope digital camera system (MC170 HD, Leica) connected to the microscope. The micrographs were edited using Photoshop Elements 3.0 (Adobe) for constructing micrographic figures (S1–S7 Figs in S1 File). Some formalin-fixed materials were also observed for identification of nematodes to the genus or family-level, and some typological characters were noted and illustrated using a camera-lucida drawing system (S8 Fig in S1 File).

## Results

### Sample collection

Fig fruits (syconia) were successfully collected from 16 out of 25 South African native *Ficus* species. Nematode DNA sequences were obtained from 14 fig species. However, because of shortages in the number of collected nematodes or material condition, only morphological vouchers were obtained from two of the fig species. Collection information and molecular identification (genotyping) are summarized in Table 1 and S1 and S2 Tables in S2 File.

### Nematode identification

Nematodes were identified based on their typological characters and phylogenetic status.

Phylogenetically, three aphelenchoidid genera (*Schistonchus*, *Ficophagus* and *Bursaphelenchus*), four diplogastrid genera (*Parasitodiplogaster*, *Teratodiplogaster*, *Pristionchus* and *Acrostichus*), and an unidentified panagrolaimid species were recognized (Table 1, S1 Table in S2 File).

Three fig-associated aphelenchoidid genera previously considered *Schistonchus* s. l. (*Schistonchus*, *Martininema* and *Ficophagus*) are typologically similar to each other [3]. Therefore, the identification of these genera was confirmed using molecular phylogenetic analyses. The phylogenetic analyses of three loci suggested that the sequences belonging to subfamily Aphelenchoidinae are separated into four *Schistonchus* spp. and nine (or ten) *Ficophagus* spp. (Figs 2–4, Table 2).

**Table 2:** Number of nematode species collected in *Ficus* subsections.

*Ficus* section	*Ficus* subsection	Nematode group							
		Aphelenchoididae			Diplogastridae				Panagrolaimidae
		*Schistonchus* s.s.	*Bursaphelenchus*	*Ficophagus*	*Parasitodiplogaster*	*Teratodiplogaster*	*Pristionchus*	*Acrostichus*	
*Galoglychia*	*Chlamydodorae*	1	-	5	3	-	-	-	1
	*Platyphyllae*	3	-	2	3	-	-	-	-
	*Galoglychia*	-	-	1		-	-	-	-
	*Calocarpe*	-	-	-	1	-	-	-	-
*Urostigma*	*Urostigma*	-	-	-	1	-	-	-	-
*Sycomorus*	*Sycomorus*	-	1	1		3	2	3	-

These are all considered to be new nematode species and were isolated from various sections/subsections of figs in South Africa, namely subgenus *Urostigma* and section *Galoglychia* figs from three subsections (*Chlamydodorae*, *Galoglychia* and *Platyphyllae*) as well as the subgenus *Sycomorus* for *Ficophagus* spp. and subgenus *Urostigma* and section *Galoglychia* figs from two subsections (*Chlamydodorae* and *Platyphyllae*) for *Schistonchus* spp. However, their phylogenetic groupings were not clearly associated with host fig subsections when put into a global context (Figs 2–4).

Diplogastrid nematodes were molecularly separated (clustered) into eight *Parasitodiplogaster* spp. (*P*. *sycophilon*, *P*. *salicifoliae*, and six new species), three *Teratodiplogaster* spp. (*T*. cf. *martini* and two new species), two *Pristionchus* spp. (*P*. *sycomori* and *Pristionchus* sp. 35), and three new *Acrostichus* spp. (Figs 5–7).

*Parasitodiplogaster* spp. were recovered from various figs in the subgenus *Urostigma* and phylogenetic groupings were in accordance with specific sections and subsections of host figs in South Africa, i.e., *P*. *salicifoliae* was isolated only from section *Urostigma* and subsection *Urostigma* figs whereas *P*. *sycophilon* and the other six new *Parasitodiplogaster* species were associated with section *Galoglychia* figs from three subsections (*Caulocarpae*, *Chlamydodorae* and *Platyphyllae*) (Figs 2–4). This contrasts with *Pristionchus*, *Teratodiplogaster* and *Acrostichus* which were found exclusively from figs in the subgenus and section *Sycomorus* and each was inferred as being monophyletic within each genus (Figs 2–4). Because we observed only one species of *Parasitodiplogaster* (n. sp. 2) associated with sycones of *F*. *burtt-davyi* from South Africa it is quite possible that it is conspecific with the species studied for its fig and fig wasp host dynamics by Jauharlina et al. [16].

The typological characters observed in DESS- and formalin-fixed materials are mostly in accordance with the phylogenetic status, i.e., the examined characters were basically identical to the generic characters or specific characters of nominal species, although some parts, e.g., male genital papillae, were sometimes not clear (S1–S8 Figs in S1 File).

In addition, a short fragment of LSU with ca 400 bp of an unidentified panagrolaimid species was recognized from *F*. *petersii* Warburg. However, further identification or phylogenetic analysis were not conducted for the nematode because the sequences closest to it were *Macrolaimus* spp. (Panagrolaimomorpha) with only 80% identity match.

## Discussion

### Taxonomic issues of fig-associated nematodes

Martin et al. [1] highlighted the potential for amazing diversity in the fig and fig wasp associated nematode fauna of Africa and the world with a survey of figs from Zimbabwe in southern Africa. The traditional typological/morphological approaches available at that time would have led to misinterpretations about this diversity and their life histories, especially in the subgenus *Sycomorus* clade figs where it appears that they observed the most diversity.

First, there was the issue of extremes in polyphenism of up to five morphotypes per genotype for a clade of, at that point, unknown subgenus *Sycomorus* fig associated *Pristionchus* species [8]. Within two *Pristionchus* spp. found in the present study, *Pristionchus* sp. 35 is considered conspecific to “*Parasitodiplogaster*” *doliostoma*, because although *Pristionchus* sp. 35 has not been formally described, it is phylogenetically close to *P*. *borbonicus* and *P*. *sycomori*, and the stomatal morphology of “*P*. *doliostoma*” is clearly similar to that of the type IV morph of these two *Pristionchus* spp. [8, 15], i.e., “*P*. *doliostoma*” is hypothesized as one morphotype of *Pristionchus* sp. 35. The species will need to be transferred to *Pristionchus*, but a formal taxonomic revision will be presented elsewhere. This polymorphism helps explain the misclassification of “*Parasitodiplogaster*” *doliostoma* by Kanzaki et al. [15] from original preserved specimens from Martin et. al [1]. Data presented herein and in Susoy et al. [8] for *Pristionchus* sp. 35 (= *Parasitodiplogaster* cf. *doliostoma*) and *P*. *sycomori* demonstrates that about half of the nematode species diversity Martin et al. [1] observed in each *Sycomorus* fig (i.e., *F*. *sur* and *F*. *sycomorus*) was probably due to a single species of *Pristionchus* that can manifest in morphotypes as divergent as five different diplogastrid genera, presumably to fill niches during the phenology of the developing syconium [8]. There is also the newly documented situation of extreme morphological divergence in *Acrostichus ziaelasi* Kanzaki, Liang, Chiu & Li from a tenebrionid beetle termitiphile [36] which converges in trophic morphology on another genus (*Rhabditolaimus*) which might help explain Martin et al.’s [1] observation of nematodes that looked like members of Cylindrocorpidae (= *Rhabditolaimus*) from *Sycomorus* figs. Given that *A*. *ziaelasi* is close to *Acrostichus* n. spp. 1 and 2 as well as three Asian *Sycomorus* fig clade associates (Figs 5 and 6) it is possible that some odd manifestation of trophic morphology in this genus of *Sycomorus* associated nematodes is yet to be observed. *Parasitodiplogaster* was reported from *F*. *sycomorus* in Susoy et al. [8] but was not recovered from *Sycomorus* figs in this study. *Teratodiplogaster* (i.e., *T*. cf. *martini* and *Teratodiplogaster* n. spp. 1 and 2) was delimited to *Sycomorus* figs and interestingly this clade was sister to the *P*. *salicifoliae* clade of nematodes from *Urostigma*/*Urostigma* figs (Fig 6).

Second, and in contrast to the intraspecific morphological diversity in *Pristionchus* spp., fig and fig wasp associated aphelenchoidids show highly conserved (convergent) morphology which resulted for many years in the lumping of three disparate genera into *Schistonchus sensu latu* which have now been separated into *Schistonchus s*.*s*., *Martininema* and *Ficophagus* [3]. In addition, there is another aphelench lineage (Parasitaphelenchinae), i.e., a clade of *Sycomorus* fig associated *Bursaphelenchus* (e.g., *Bursaphelenchus* n. sp. 1 and *B*. *sycophilus*) (Figs 2 and 3, S2 Fig in S1 File) that morphologically converged on the mouth form (stylet) motif of *Schistonchus s*.*l*. This makes aphelenchoidid nematodes difficult to separate from typical *Schistonchus s*.*l*. especially in mixed populations of fig-associated nematodes [3, 27, 29]. For example, only one aphelenchoidid, *S*. *africanus* has been described from the studied region from *F*. *burkei* [13], and thus, one of the six aphelenchoidids (five *Ficophagus* and one *Schistonchus* spp.) recovered from *F*. *burkei* in this study could be conspecific to *S*. *africanus*. However, because of the typological similarity among fig-associated aphelenchoidids and the lack of detailed morphological and genetic information in the original description [9, 13], we could not resolve or further characterize *S*. *africanus* using current taxonomic standards. In addition, the number and quality of materials collected in the present study were not sufficient to characterize each genotype typologically. Therefore, the conspecificity and the phylogenetic status of *S*. *africanus* with any of the *F*. *burkei* isolates in this study remains unclear and will require a reverse taxonomy approach. In addition, a species of *Bursaphelenchus* which is closely related to *B*. *sycophilus* isolated from *F*. *variegata* in Japan (Figs 2 and 3) [27] was isolated from *F*. *sur* from Pretoria corroborating a subgeneric *Sycomorus* fig co-association for this fig-derived *Bursaphelenchus* clade. Susoy et al. [8] reported discovery of a *Bursaphelenchus* from *F*. *sycomorus* from Pretoria that will require further work for phylogenetic placement.

Lastly, there is the issue of mixed lineages and incomplete lineage sorting from movement of species of nematodes among the different pollinators or inquilines that might visit different *Ficus* species [37, 38]. Clearly, Martin et al. [1] made an extraordinary discovery about fig-associated nematodes in Africa, especially when it comes to *Sycomorus* clade figs, which is corroborated and further elucidated here with the aid of molecular inferences (Figs 2–7).

In addition to the two typical fig-associated families (Aphelenchoididae and Diplogastridae), an unidentified panagrolaimid was recognized in the present study. The precise phylogenetic status of the panagrolaimid is still unknown. This finding suggests that nematode colonization in fig syconia has occurred many times, more than we expected, and further diversity of fig associated nematodes will be discovered by more extensive collecting.

### Host fig association

The phylogenetic relationships inferred for the fig-associated aphelenchoidid genera (*Schistonchus* and *Ficophagus*) recovered in this study were not clearly associated with geography or host fig subsections (Figs 2–4). The only true affiliation between *Ficophagus* and fig lineages appears to be between Central American delimited *Pharmacosycea* figs and their associated nematodes (both *Ficophagus* and *Parasitodiplogaster*), but in the South African figs, *Ficophagus* n. sp. 4 ex *F*. *sur* appeared consistently monophyletic with other *Ficophagus* spp. from *Sycomorus* clade figs from other continents in SSU and LSU inferences (Figs 2 and 3). Similarly, the discovery of *Bursaphelenchus* sp. 1 from *F*. *sur* from Pretoria and its monophyly with *B*. *sycophilus* from *F*. *variegata* from Japan [27] establishes a subgeneric *Sycomorus* fig co-association for a fig-adapted *Bursaphelenchus* clade. It also portends that the *Bursaphelenchus* reported from *F*. *sycomorus* by Susoy et al. [8] will be monophyletic with other *Sycomorus* clade fig associated *Bursaphelenchus* from Africa and Australasia when re-examined.

Poinar [12] described *P*. *sycophilon* as the first member of this genus from *F*. *burkei* (subsection *Chlamydodorae*) from Zimbabwe without molecular data. We can now place *P*. *sycophilon* with six new *Parasitodiplogaster* species that are all associated with *Urostigma* subgenus, section *Galoglychia* figs from three subsections (*Caulocarpae*, *Chlamydodorae* and *Platyphyllae*) which form an African delimited *Parasitodiplogaster* clade that is associated with an African delimited *Ficus* clade (Figs 5–7). In contrast, *P*. *salicifoliae* was isolated only from section *Urostigma* and subsection *Urostigma* figs from South Africa (*F*. *salicifolia* and *F*. *ingens*) but formed a monophyletic clade with other *Parasitodiplogaster* species from this clade of figs from Australia (i.e., *P*. *australis* Bartholomaeus, Davies, Ye, Kanzaki & Giblin-Davis) and China (i.e., *P*. *religiosae* Zeng, Zeng, Zhang, Ye, Cheng, Kanzaki & Giblin-Davis) (Figs 5 and 6).

Multiple lineages of a nematode genus were found in the same fig species, and these lineages included close relatives (a pair of sister species), in the present study. For example, *F*. *burkei* harbored two *Parasitodiplogaster* spp. (*P*. *sycophilon* and its close relative, *Parasitodiplogaster* n. sp. 6), and five *Ficophagus* spp. (*Ficophagus* n. spp. 1, 2, 5–7, where sp. 1 and sp. 2 are close to each other, and spp. 5–7 form a subclade), and *F*. *sur* had three or four closely related *Acrostichus* spp. (n. sp. 1–3 or 4), *T*. cf. *martini*, *Pristionchus* sp. 35 (= *Parasitodiplogaster* cf. *doliostoma*) and *Ficophagus* sp. 4 (Figs 2–7, Table 1, S1 Table in S2 File). Similar multiple colonization events and duplications of the lineages were found in previous studies, e.g., *F*. *hispida* L. harbors two *Ficophagus* spp. (*F*. *centerae* (Zeng, Giblin-Davis & Ye) and an undescribed *Ficophagus* species), three *Martininema* spp. (*M*. *guangzhoensis* (Zeng, Giblin-Davis & Ye), *M*. *baculum* (Davies, Bartholomaeus, Kanzaki, Ye & Giblin-Davis) and an undescribed *Martininema* species), and an undescribed *Schistonchus* sp. [39–41], and *F*. *maxima* Mill. has three *Parasitodiplogaster* spp. (*P*. *maxinema* Poinar & Herre, *P*. *pharmaconema* Kanzaki, Giblin-Davis, Ye, Herre & Center and an undescribed species which are all close to each other) and *Ficophagus maxima* Davies, Ye, Center, Kanzaki, Bartholomaeus, Herre, Esquivel & Giblin-Davis [42–44].

Multiple colonization events and the duplication of lineages are also well documented in wood and bark beetles and their associated nematodes. A species of bark beetle often harbors several different lineages of aphelenchoidids and diplogastrids [45, 46], and two closely related *Bursaphelenchus* spp. (*B*. *luxuriosae* Kanzaki & Futai and *B*. *acaloleptae* Kanzaki, Ekino, Maehara, Aikawa & Giblin-Davis) share the same host (Araliaceae trees) and carrier beetle (*Acalolepta luxuriosa* (Bates)) [47].

The wasp associations of the nematodes, e.g., host range, were not examined in the present study. These nematodes are carried by fig wasps, and thus, their fig host range is determined by the wasps. Several previous authors reported that host specificity of the fig wasps which is determined by chemical signals from the fig host can become less stringent. One species of wasp can be associated with multiple species of figs, and vice versa [37, 38, 48, 49]. In addition, the host switching, duplication of the lineages, and extinction and fill-in of the wasp lineages can drive the diversification of figs and their pollinating wasps [49, 50]. Because the nematodes are tightly associated with figs and fig wasp as the habitat/host and carrier/host, their diversification and host switching are likely to be affected by those of the carrier host. Further collections of figs, wasps and nematodes and specifying their host/carrier ranges, i.e., carrier wasp range of nematodes and host range of wasps, are necessary to understand their tripartite associations.

In the present study, we focused on the diversity of nematodes in relation to their host/habitat fig lineages but did not conduct extensive research involving the diversity and host range of the fig wasps. Further studies on biological and phylogenetic relationships among nematodes, fig wasps and fig trees will help reveal the mechanism(s) and give further insights into the evolutionary context of these tripartite relationships.

Our study has expanded and improved the clarity and resolution of Martin et al. [1] with a more extensive spatial/temporal snapshot of the fig-associated nematode fauna from South Africa with the aid of molecular technology. However, it still relied upon the luck of finding asynchronously fruiting fig trees in the proper phases with nematodes in good condition with different association rates with their fig wasp and/or fig hosts from a large area during a finite survey. Thus, there are potentially many more nematode species and interesting life history discoveries to be made to add upon what is reported here. There is also much reverse taxonomy needed to finish tying the genotypes reported in this study with well-defined morphotypes and biological/life history traits for South African species of fig associated nematodes as recently initiated by Wöhr et al. [17, 18] and Susoy et al. [8].

## Supporting information

S1 File Typological characters of several nematode species recovered from the fig materials. Light micrographs or drawn are provided as supplementary figures.
(PDF)

S2 File Detailed collection information and taxonomic status of *Ficus* species appeared in the present study. (XLSX)
