# Supplementary material for: Molecular diversity and relationships of fig associated nematodes from South Africa
Source: PLoS One. 2021 Aug 10;16(8):e0255451. doi: 10.1371/journal.pone.0255451 (PMC8354458; doi:10.1371/journal.pone.0255451)
Supplement: S1 File — Light micrographs or drawn are provided as supplementary figures. (PDF) [file pone.0255451.s001.pdf]

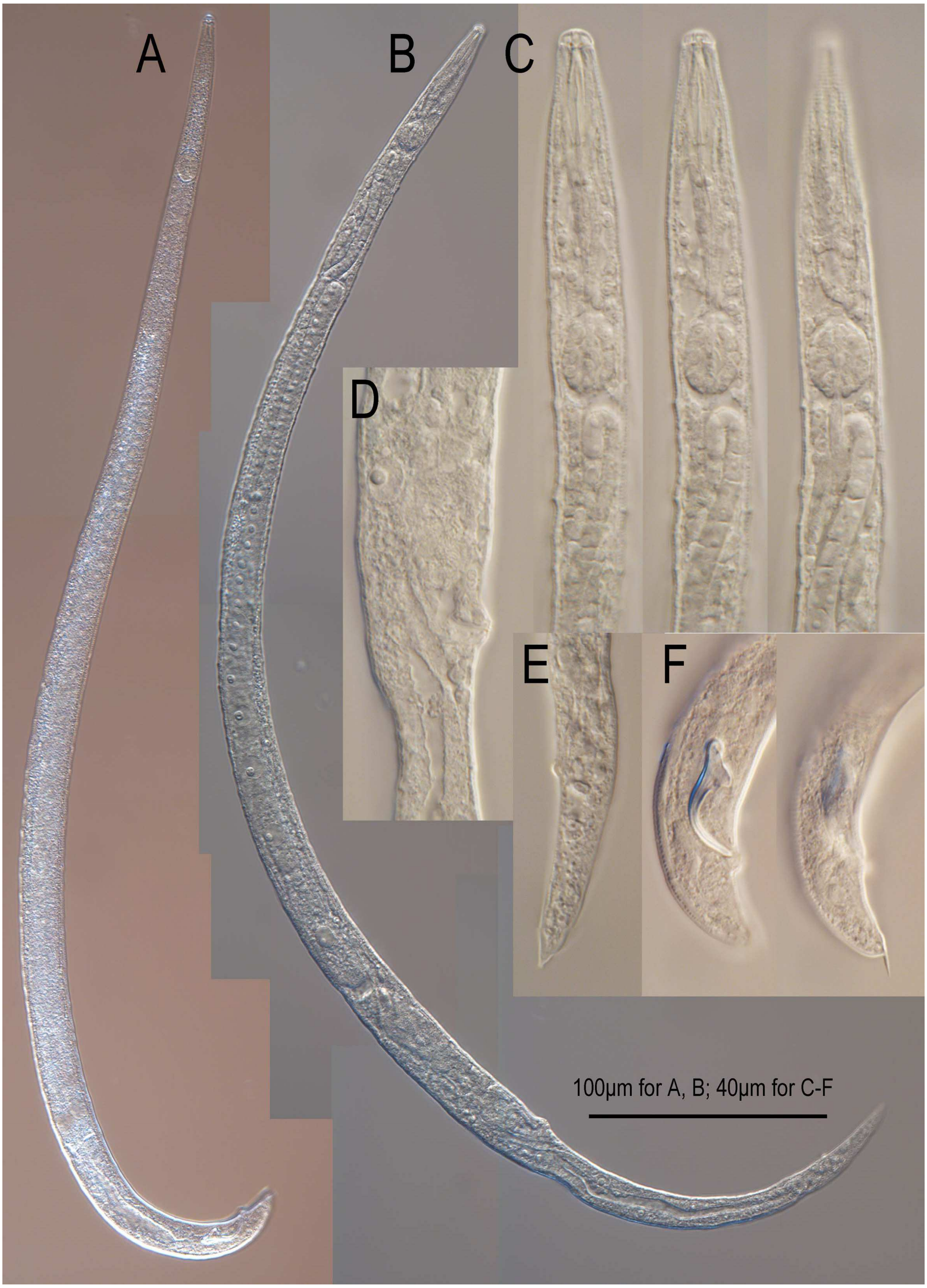

S1 Figure. *Ficophagus* n. sp. 4 recovered from *Ficus sur* from Pretoria, S.A. A: Male; B: Female; C: Anterior region of female in three different focal planes; D: Vulval region of female; E: Female tail; F: Male tail in two different focal planes.

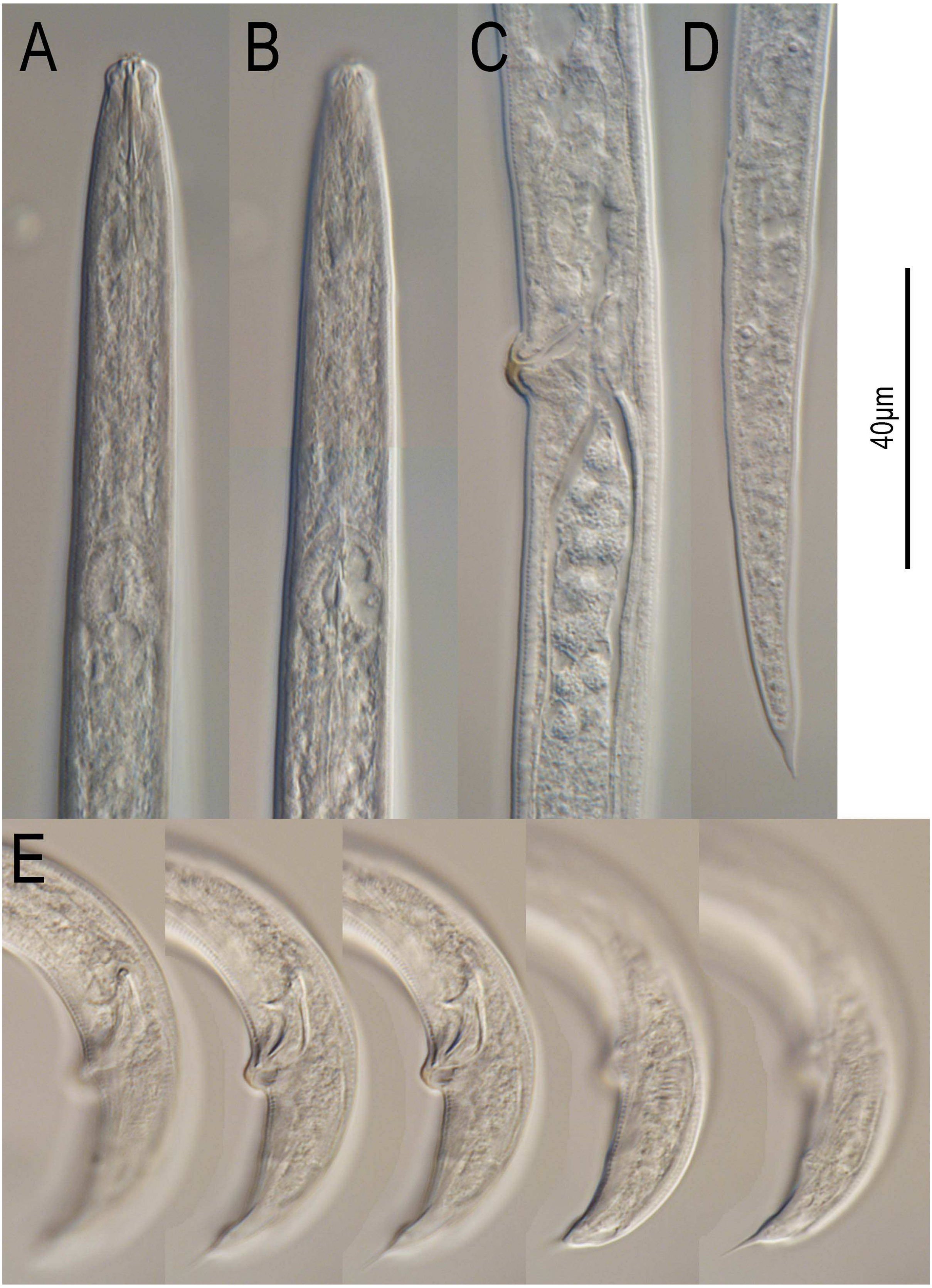

S2 Figure. *Bursaphelenchus* n. sp. 1 recovered from *Ficus sur* from Pretoria, S.A. A, B: Anterior region in two different focal planes; C: Vulval region of female; D: Female tail; E: Male tail in five different focal planes.

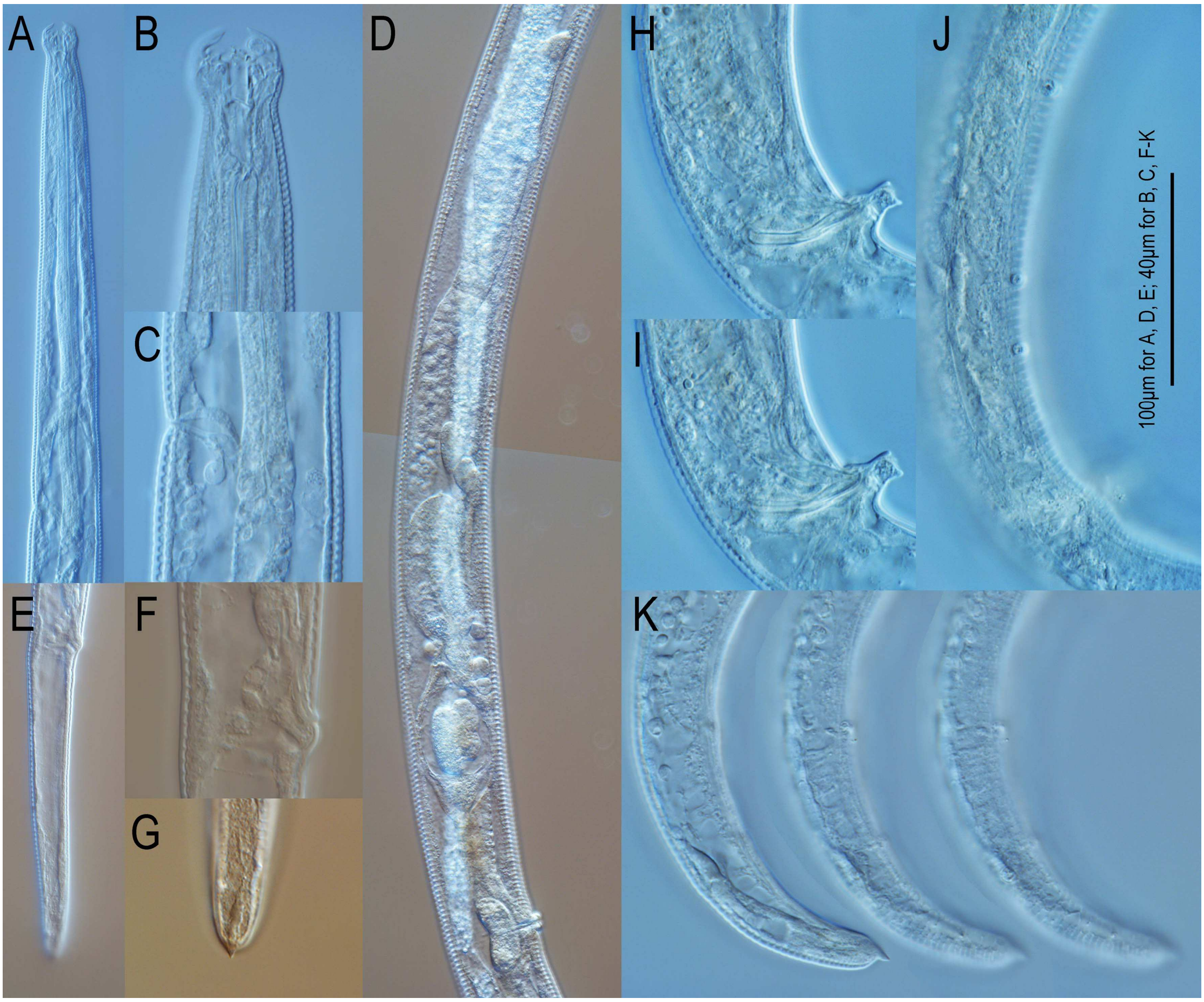

S3 Figure. ***Teratodiplogaster*** **cf.** ***martini*** **recovered from** ***Ficus sur*** **from Pretoria, S.A.**
A: Whole pharyngeal region; B: Stomatal region; C: Vulval region; D: Anterior gonad of female; E: Whole female tail; F: Anus and rectum of female; G: Female tail tip; H, I: Cloacal region in two different focal planes; J: Anterior part of male tail; K: Posterior part of male tail in three different focal planes.

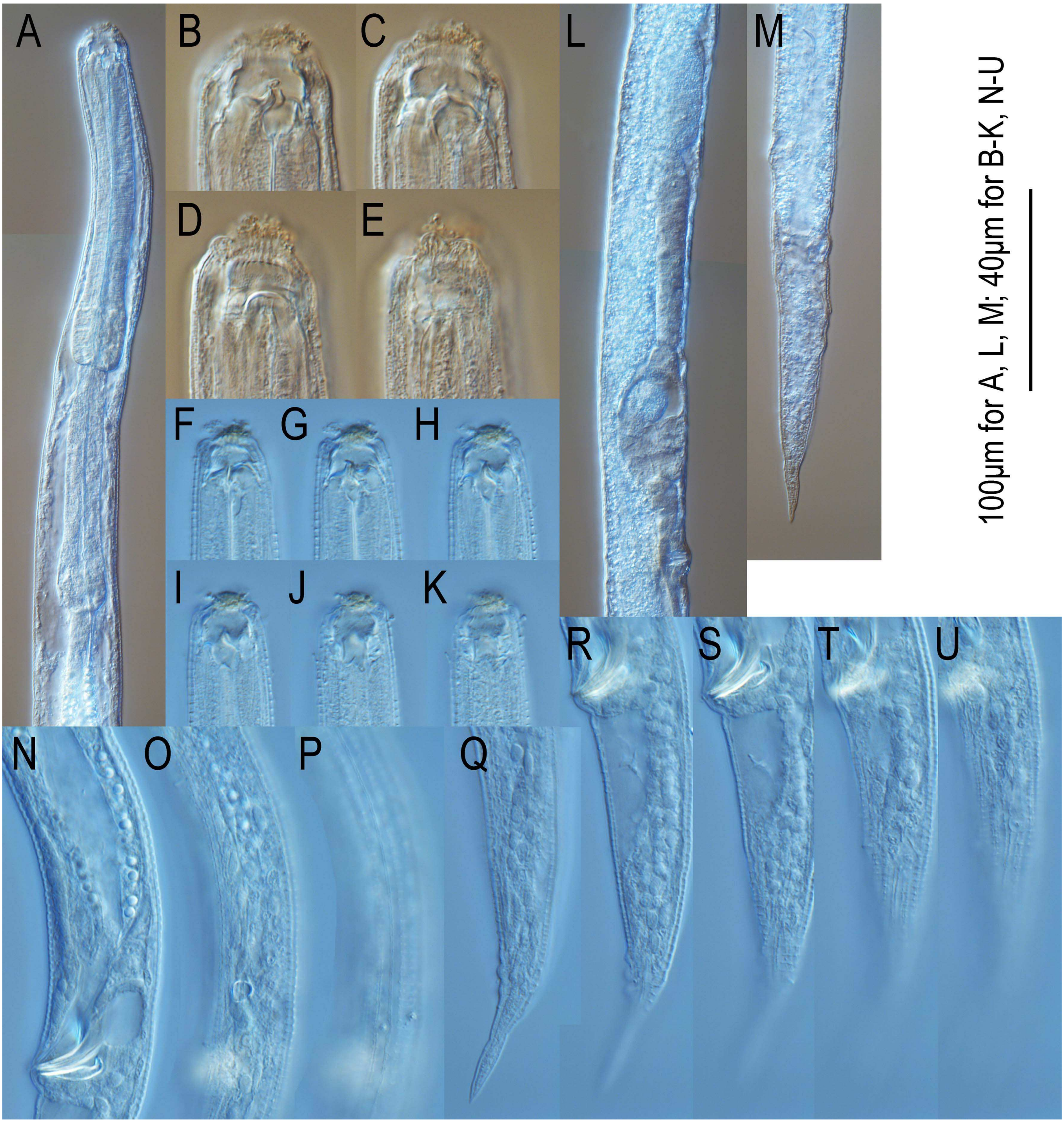

S4 Figure. ***Pristionchus*** **sp. 35 (Susoy et al., 2016) recovered from** ***Ficus sur*** **from Pretoria, S.A.** A: Whole pharyngeal region; B-E: Stomatal region of type I individual in four different focal planes; F-K: Stomatal region of type III individual in six different focal planes; L: Anterior gonad of female; M: Female tail; N-P: Anterior part of male tail in three different focal planes; Q-U: Posterior part of male tail in five different focal planes.

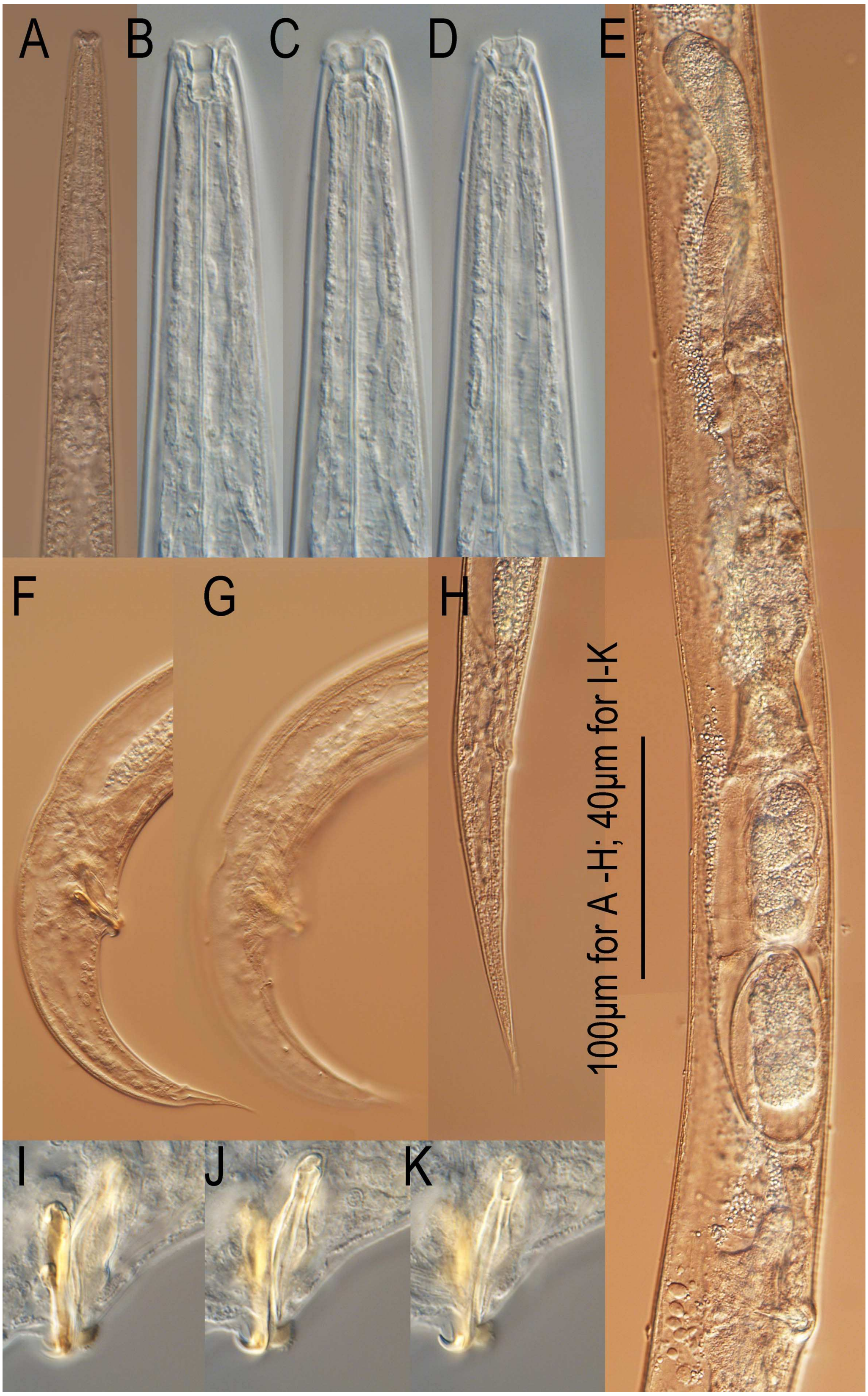

S5 Figure. *Acrostichus* n. sp. 1. recovered from *Ficus sur* from Pretoria, S.A. A: Whole pharyngeal region; B-D: Anterior pharynx region in three different focal planes; E: Anterior gonad of female; F, G: Male tail in two different focal planes; H: Female tail; I-K: Spicule and gubernaculum of male in three different focal planes.

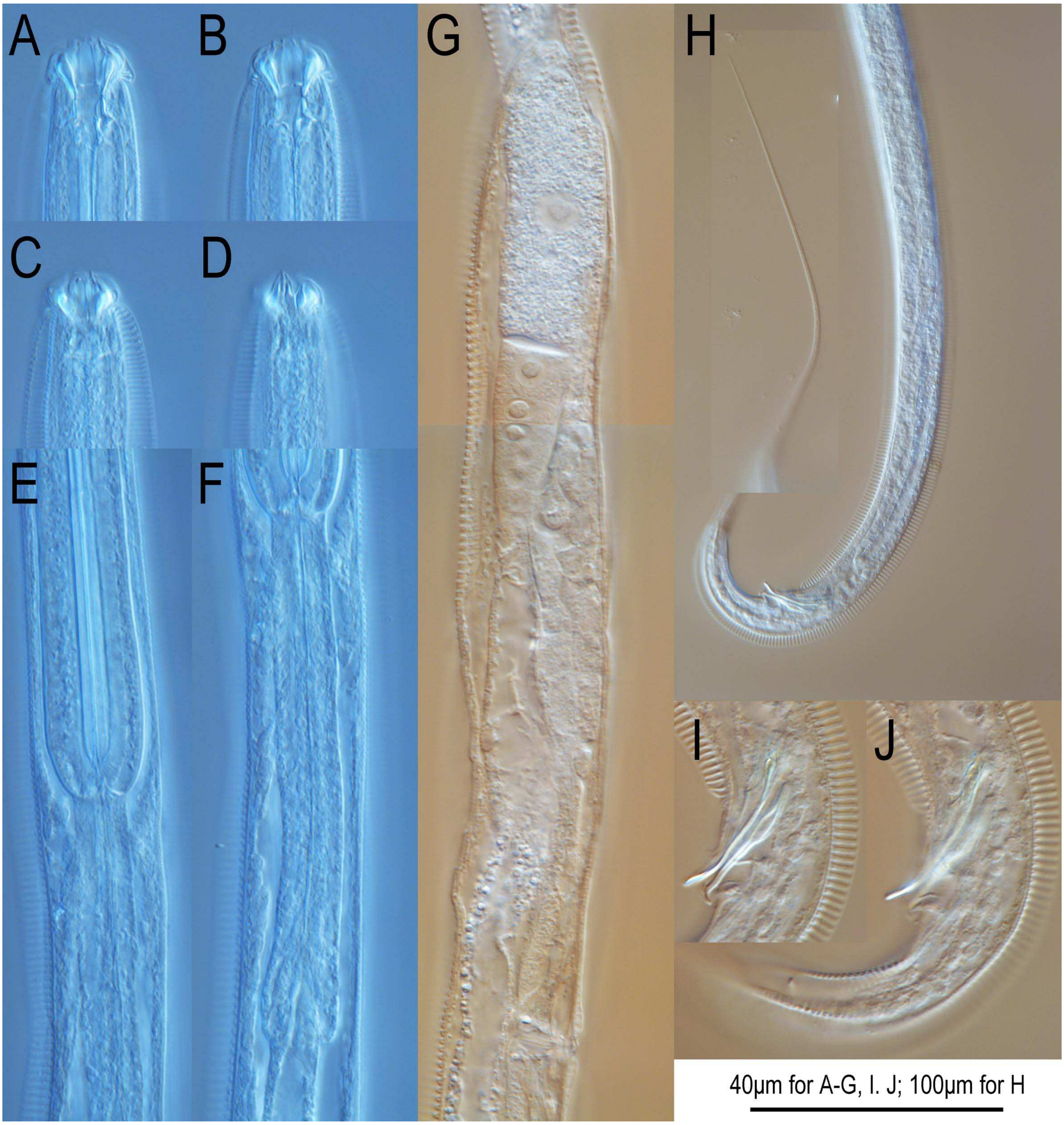

S6 Figure. ***Acrostichus*** **n. sp. 2. recovered from** ***Ficus sur*** **from Pretoria, S.A.** A-D: Stomatal region in four different focal planes; E, F: Anterior and posterior pharynx; G: Anterior gonad of female; H: Posterior body of male; I, J: Spicule and gubernaculum of male in two different focal planes.

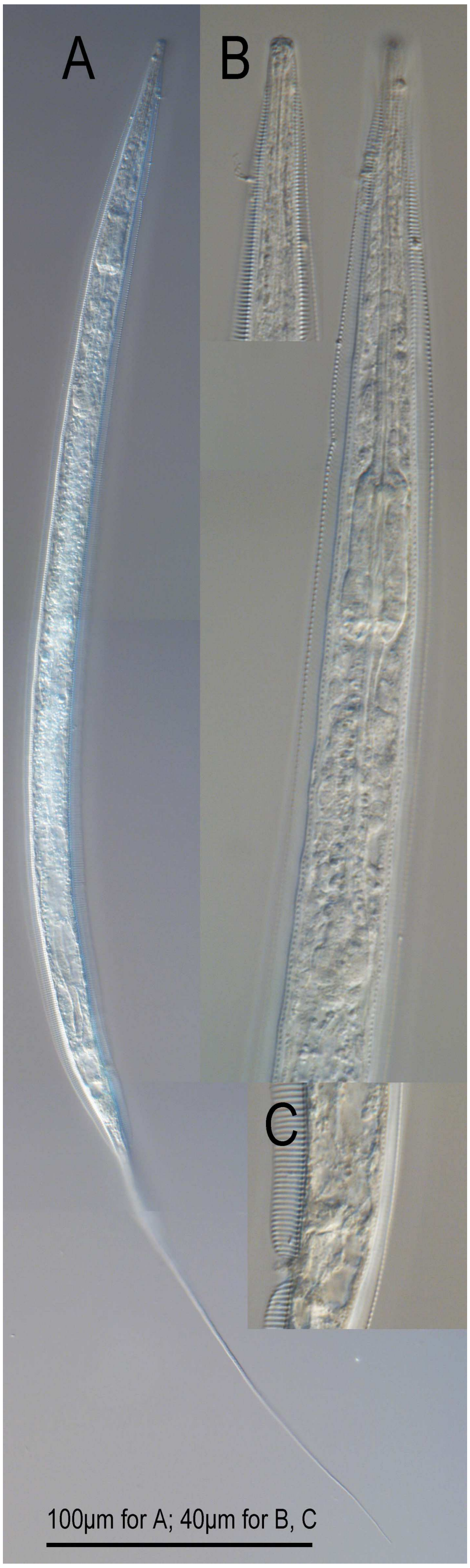

S7 Figure. Unidentified diplogastrid juvenile recovered from *Ficus sur* from Pretoria, S.A. A: Whole body; B: Anterior region in two different focal planes; C: Anal region. Because the PCR amplification for molecular identification was not successful, further species identification was not conducted.

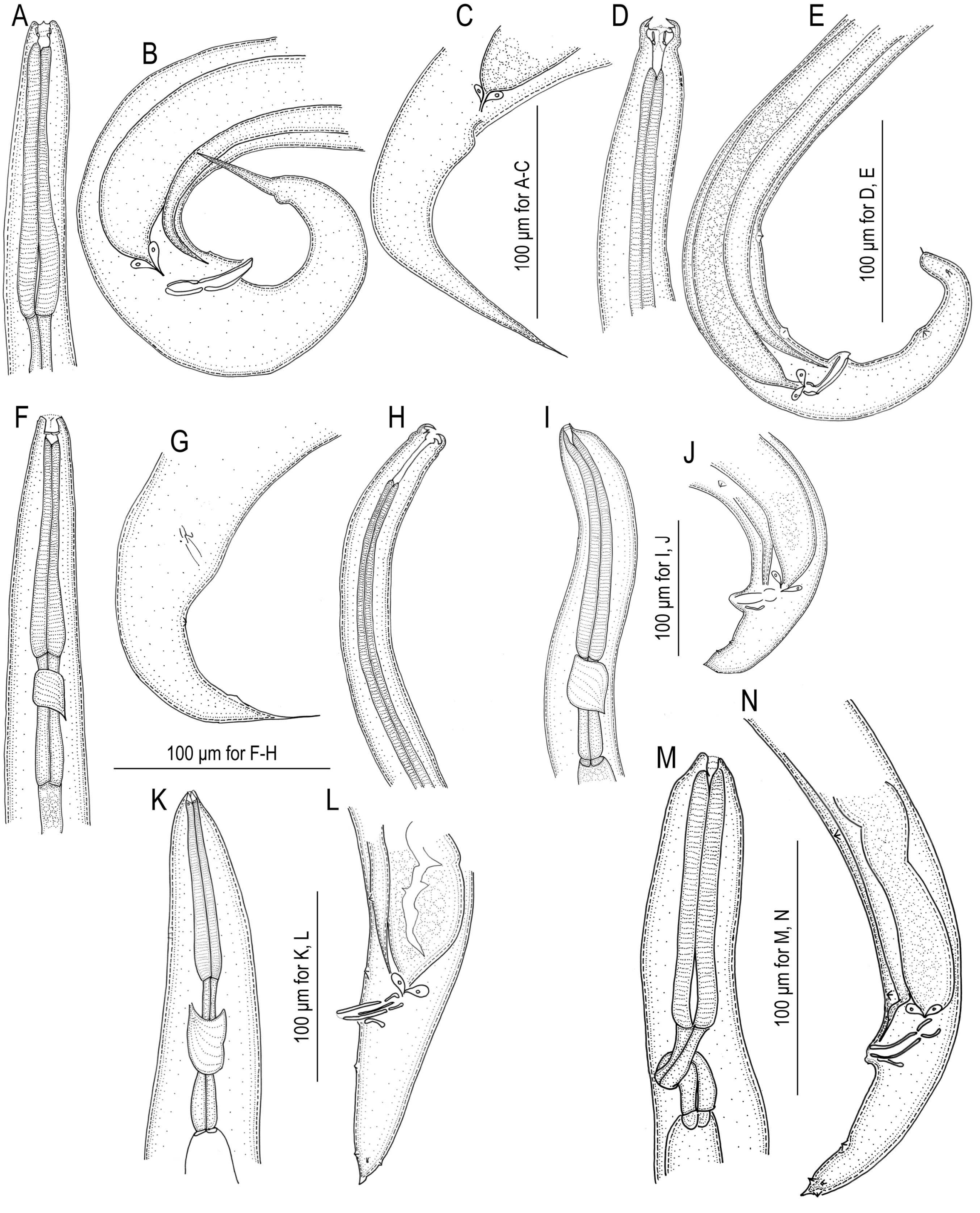

S8 Figure. Typological illustrations of formalin-fixed materials. Either pharyngeal region, mail tail or female tail regions are drawn. Species status are estimated from the host Ficus species and the results from molecular identification. A-C: *Acrostichus* n. sp. 1 recovered from *F. sur*; D, E: *Teratodiplogaster* cf. *martini* recovered from *F. sur*; F. G: *Pristionchus sycomori* with type IV stoma recovered from *F. sycomorus*; H: *Teratodiplogaster* n. sp. 1 recovered from *F. sycomorus*; I, J: *Parasitodiplogaster* n. sp. 3 recovered from *F. abutilifolia*; K, L: *Parasitodiplogaster* n. sp. 5 recovered from *F. stuhlmannii*; M, N: *Parasitodiplogaster* n. sp. 2 recovered from *F. burtt-davyi*.
